# Farnesol, a Potential Efflux Pump Inhibitor in *Mycobacterium smegmatis*

**DOI:** 10.3390/molecules15117750

**Published:** 2010-10-29

**Authors:** Jing Jin, Ji-Yu Zhang, Na Guo, Hui Sheng, Lei Li, Jun-Chao Liang, Xue-Lin Wang, Yang Li, Ming-Yuan Liu, Xiu-Ping Wu, Lu Yu

**Affiliations:** 1 Key Laboratory of Zoonosis Research, Ministry of Education, Institute of Zoonosis, College of Animal Science and Veterinary Medicine, Jilin University, Changchun, 130062, China; 2 Key and Open Laboratory of Veterinary Pharmaceutical Engineering, Lanzhou Institute of Animal Science and Veterinary Pharmaceutics, Chinese Academy of Agricultural Sciences, Lanzhou, 730050, China; 3 Laboratory of Nutrition and Functional Food, Jilin University, Changchun, 130062, China; 4 The First Hospital of Jilin University, Jilin University, Changchun, 130021, China

**Keywords:** farnesol, inhibitor, efflux pump, *Mycobacterium smegmatis*

## Abstract

The active multidrug efflux pump (EP) has been described as one of the mechanisms involved in the natural drug resistance of bacteria, such as mycobacteria. As a result, the development of efflux pumps inhibitors (EPIs) is an important topic. In this study, a checkerboard synergy assay indicated that farnesol both decreased the minimum inhibitory concentration (MIC) of ethidium bromide (EtBr) 8-fold against *Mycobacterium smegmatis* (*M. smegmatis*) mc^2^155 ATCC 700084 when incorporated at a concentration of 32 μg/mL (FICI = 0.625) and decreased MIC 4-fold at 16 μg/mL (FICI = 0.375). Farnesol also showed synergism when combined with rifampicin. A real-time 96-well plate fluorometric method was used to assess the ability of farnesol to inhibit EPs in comparison withfour positive EPIs: chlorpromazine, reserpine, verapamil, and carbonyl cyanide *m*-chlorophenylhydrazone (CCCP). Farnesol significantly enhanced the accumulation of EtBr and decreased the efflux of EtBr in *M. smegmatis*; these results suggest that farnesol acts as an inhibitor of mycobacterial efflux pumps.

## Introduction

Some mycobacteria are pathogens that can cause significant morbidity and mortality; among these, *Mycobacterium tuberculosis* (*M. tuberculosis*) is one of the oldest and most common causes of infection and death in the World, *Mycobacterium avium* (*M. avium*) often causes blood infection in AIDS patients, and *Mycobacterium smegmatis* (*M.* *smegmatis*) is also an opportunistic pathogen [1]. The 13th annual tuberculosis (TB) report from the World Health Organization (WHO; published on World TB Day, March 24, 2009) revealed that there were an estimated 9.27 million new cases of tuberculosis worldwide in 2007 [2]. With the emergence of mycobacteria expressing multidrug resistance (MDR), there is an urgent need for new antituberculosis drugs to treat TB [3].

Membrane-based efflux pump (EP) systems play an important role in bacterial pathogenicity and antimicrobial resistance in bacteria. Recent reports have shown that efflux pumps (EPs) can decrease intracellular drug concentrations used in a clinical setting and that changes in the expression of EPs can rapidly result in antibiotic resistance [4] by preventing the compounds from reaching their intended targets [5,6]. Effective bacterial EP inhibitors (EPIs) should decrease the intrinsic resistance of bacteria to antibiotics, reverse acquired resistance and reduce the frequency of the emergence of newly resistant mutant strains [7]. To our knowledge, few EPIs for mycobacteria have been identified so far [8]. Even though several experimental compounds, such as reserpine, chlorpromazine, verapamil and carbonyl cyanide *m*-chlorophenylhydrazone (CCCP), have been shown to have EPI effects against mycobacteria both *in vitro* and *ex vivo* [8,9], these compounds have not yet fulfilled certain requirements of clinical relevancy (*i.e.*, serum concentration, toxicity, immunosuppression, and stability and solubility concerns) in human or veterinary medicine [10,11]. Therefore, it is important to explore new EPIs for mycobacteria.

**Figure 1:**
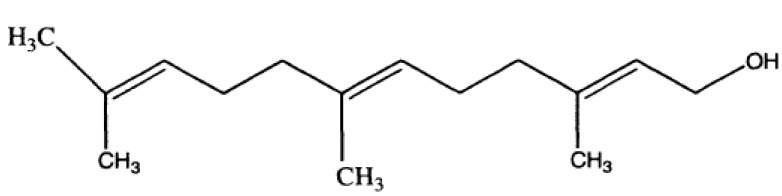
Chemical structure of farnesol.

Recent reports have shown that the 15-carbon isoprenoid component farnesol (Figure 1), a natural plant metabolite, can intensify the effect of antimicrobial agents on *Staphylococcus aureus* (*S. aureus*) and *Escherichia coli* (*E. coli*) [12,13]. These studies demonstrated that farnesol inhibits oxidation-reduction reactions and also increases the susceptibility of *S. aureus* to gentamicin; because gentamicin requires ATP-dependent transport to enter the cells, this result suggests that membrane integrity is disrupted by farnesol [14]. 

Membrane damage would also facilitate the penetration of antibiotics, such as macrolides and quinolones [13]. Farnesol is likely to be potent, providing effective K^+^ leakage from the cytoplasm and causing less toxicity in humans [15]. These interesting results led us to investigate whether farnesol exhibits EPI activity in mycobacteria.

Ethidium bromide (EtBr) is a biocompatible molecule that neither affects cell viability nor perturbs cellular functions under defined and controlled conditions; it is also a well-known substrate of many EPs in various multidrug-resistant microorganisms, including *S. aureus* and mycobacteria [10,16,17]. *Mycobacterium smegmatis* (*M. smegmatis*) is nonpathogenic, requiring less stringent containment facilities, and it grows at a relatively high rate in many defined and nutrient-restricted media [18]. Therefore, it is advantageous to use EtBr and *M. smegmatis* collaboratively to study EPI activity. In this study, we used a real-time 96-well plate fluorometric method that has recently been described [9,19], employing a modification in order to evaluate the inhibiting effects of farnesol on the *M. smegmatis* mc^2^155 drug efflux system.

## Results

### MIC results

MIC values of all the compounds tested are listed in Table 1. The compound farnesol showed antimycobacterial activity at a MIC of 64 μg/mL. The MICs of EtBr, chlorpromazine, reserpine, verapamil and CCCP for *M. smegmatis* mc^2^155 were 8, 32, 256, 300, and 25 μg/mL, respectively; these findings are similar to previous results [8,9]. These data indicate that the antimycobacterial activity of farnesol is better than that of the positive control EPIs reserpine and verapamil and worse than that of chlorpromazine and CCCP. The MICs of these compounds for *M. smegmatis* mc^2^155 were also later used as a reference for determining the EPI concentrations for the EP inhibition assay. The MIC of rifampicin is 64 μg/mL against *M. smegmatis* mc^2^155.

**Table 1:** MICs and checkerboard synergy of agents employed for *M. smegmatis* mc^2^155.

Compound	MIC (μg/mL)	Concentration as modulator (μg/mL)	Modulation factor (EtBr)	FICI
Farnesol	64	8	2	0.625
		16	8	0.375
		32	8	0.625
Chlorpromazine	32	8	1	1.25
		16	4	0.75
Reserpine	256	32	2	0.625
		64	4	0.5
Verapamil	300	32	1	1.107
		64	2	0.713
CCCP	25	16	2	1.14

MIC of EtBr = 8 μg/mL; Modulation factor = MIC (EtBr)/MIC (EtBr + modulator).

### Checkerboard synergy assay results

In this experiment, the checkerboard synergy assay was employed to evaluate both the synergistic interactions of farnesol, reserpine, verapamil, chlorpromazine and CCCP with EtBr and the modulation of EPIs on the MIC of EtBr against *M. smegmatis* mc^2^155 ATCC 700084. The results are listed in Table 1. The FICI between farnesol at 16 μg/mL and EtBr at 1 μg/mL was 0.375, indicating a good synergism, whereas the FICIs between chlorpromazine, verapamil, CCCP and EtBr all showed indifferent interactions (FICI = >0.5~2), and the combination of reserpine and EtBr showed a weak synergism (FICI = 0.5). In addition, the FICI between farnesol at 16 μg/mL and rifampicin at 8 μg/mL was 0.375. These results suggest that farnesol may be a potential inhibitor of efflux pump in *M. smegmatis*.

### EtBr efflux inhibition

**Figure 2:**
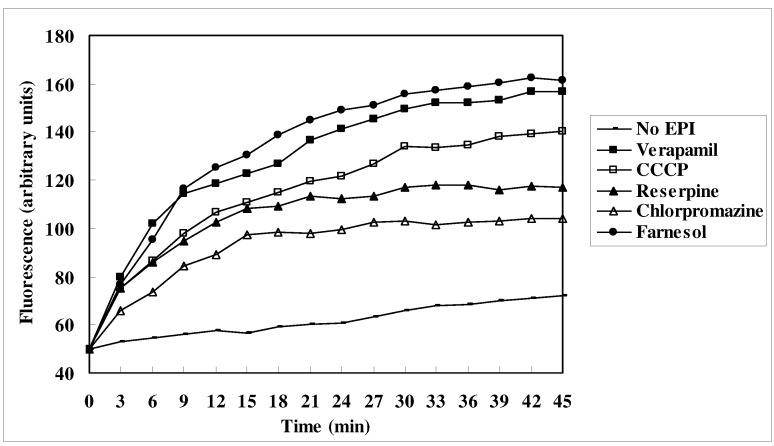
Effect of EPIs on the accumulation of EtBr in *M. smegmatis* mc^2^155 cells. Concentrations of reserpine, chlorpromazine, verapamil, CCCP and farnesol are at half their MICs (See also Table 1).

In an EtBr accumulation assay, we compared the levels of EtBr accumulation in *M. smegmatis* cells treated by reserpine, chlorpromazine, verapamil, CCCP and farnesol within 45 minutes (see Figure 2). The level of EtBr accumulation in the control group (no EPI treatment) was the lowest. However, with the addition of the positive control EPIs or farnesol, the level of EtBr accumulation in the experimental groups increased significantly. The verapamil group had the highest level of EtBr accumulation among the groups; the CCCP group showed a higher level of EtBr accumulation than the reserpine group, and the chlorpromazine group showed the lowest level of accumulation. Compared to these EPI-treated groups, the level of EtBr accumulation induced by farnesol treatment was higher than the verapamil treatment.

**Figure 3:**
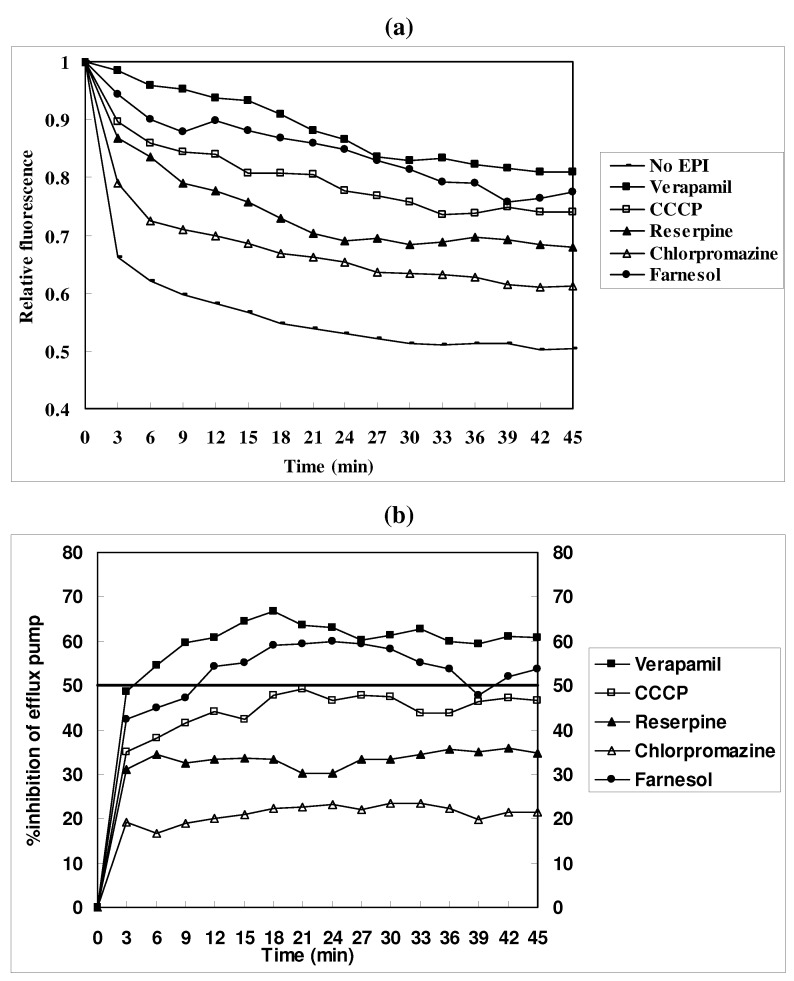
Effect of EPIs on the efflux of EtBr in *M. smegmatis* mc^2^155 cells (a) and the percentage inhibition of efflux pump by EPIs (b). The assay was conducted at 25 °C with glucose, after loading the mycobacteria with EtBr at 4 μg/mL in the presence of verapamil at half its MIC. Concentrations of reserpine, chlorpromazine, verapamil, CCCP and farnesol are at half of their MICs (Table 1). The horizontal line indicates 50% efflux inhibition in Figure 3 (b).

The results of the EtBr efflux assay of *M. smegmatis* cells within 45 minutes of treatment by reserpine, chlorpromazine, verapamil, CCCP and farnesol are shown in Figure 3. According to the results in Figure 3(a), each known EPI tested showed efflux inhibition in *M. smegmatis* mc^2^155 cells. Verapamil had the strongest ability to inhibit the activity of the efflux pump in *M. smegmatis* mc^2^155 cells relative to the other three positive EPIs. CCCP exhibited a stronger ability to inhibit the activity of the efflux pump in *M. smegmatis* mc^2^155 cells relative to reserpine. Though the chlorpromazine group showed the weakest effect, it also showed efflux inhibition activity in *M. smegmatis* mc^2^155 cells. Compared with the groups that were treated with EPIs, the efflux inhibition ability of farnesol was higher than CCCP but lower than verapamil. Mean results were expressed as the percentage inhibition of total efflux observed for *M*. *smegmatis* mc^2^155 cells in the absence of inhibitors in Figure 3 (b). 

**Figure 4:**
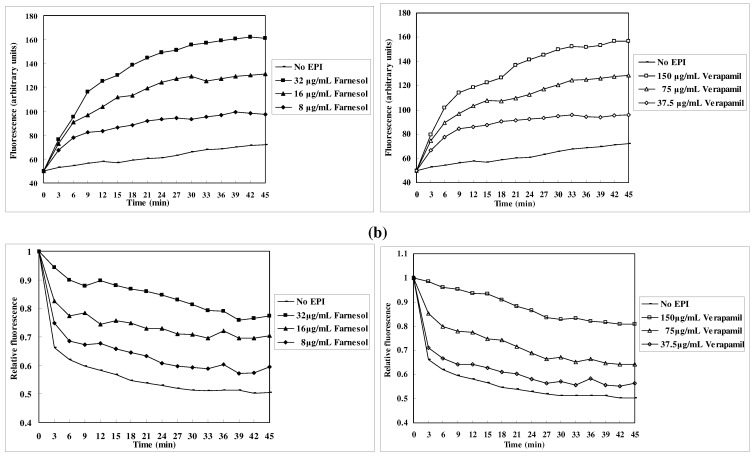
Effect of farnesol and verapamil on the accumulation (a) and efflux (b) of EtBr from *M. smegmatis* mc^2^155 cells in different concentrations.

Figure 4 showed that a higher concentration of farnesol resulted in a stronger EtBr efflux inhibitory effect in *M. smegmatis* mc^2^155 cells, indicating that farnesol acted in a concentration-dependent manner during the period of the assay.

## Discussion

Drug resistance in mycobacteria (particular multidrug-resistant strains of *M. tuberculosis*) is increasing yearly, a phenomenon that seriously threatens the ability of medicine to control TB. It is difficult to reverse this situation without ensuring that all patients are diagnosed rapidly and treated effectively so that resistant strains are not produced and transmitted in the community [20]. It has been established that the expression of EPs is one of the mechanisms involved in the natural drug resistance of mycobacteria [5]. There are five families of bacterial drug EPs [21]. Two of these are large superfamilies of ancient origin are as follows: the ATP-binding cassette (ABC) superfamily and the major facilitator superfamily (MFS); the other three are smaller families that have developed more recently: the small multidrug resistance (SMR) family, the resistance-nodulation-cell division (RND) family, and the multidrug and toxic compounds extrusion (MATE) family. These EPs can transport drugs through the bacterial envelope and limit the intracellular accumulation of toxic compounds, such as antibiotics, antimicrobial peptides, metals and detergents [11]. Therefore, there is an urgent need for novel drugs with new modes of action, such as EPIs, to prevent the rise of MDR bacteria [16]. There are currently no EPI/antimicrobial drug combinations on the market, although both academic institutions and the pharmaceutical industry are continually conducting research to identify potential EPIs [22]. Identifying EPIs from natural sources is an alternative approach.

As mentioned above, the isoprenoid component farnesol has been found to enhance the effects of antimicrobial agents on *S. aureus* and *E. coli* [12,13]. In this experiment, two different assays were performed to identify whether farnesol has the potential to act as an efflux inhibitor on *M. smegmatis* mc^2^155 possessing an EP; these assays included a checkerboard synergy assay on antimycobacterial synergism and a fluorometric method on a real-time basis. Checkerboard techniques have been widely used to evaluate the activity of antimicrobial combinations [23,24]. The real-time 96-well plate fluorometric method requires relatively small volumes of the EPIs, making it especially advantageous for testing rare or expensive compounds [9,19,25]. In the fluorometric method, fluorochrome EtBr has a low fluorescence signal outside the bacterial cell that increases once inside the cell in a concentration-dependent manner and is widely recognized as the best candidate for monitoring EP activity [9]. In the present experiments, referred to as Rodrigues *et al*. [9], four well-known EPIs (reserpine, chlorpromazine, verapamil and CCCP) were used as reference substances to evaluate the extent of possible efflux inhibition in the fast-growing mycobacterial strain *M. smegmatis* mc^2^155. According to the results of the checkerboard synergy assay, farnesol decreased the MIC of EtBr 8-fold at 32 μg/mL, indicating that farnesol was effective in blocking EtBr efflux in *M. smegmatis* and that it may be a potential inhibitor of EtBr efflux in *M. smegmatis*. Couto *et al*. demonstrated that a reduction in the MICs to at least a quarter of their original values in the presence of the EPI was considered to be indicative of efflux activity [26]. The FICI between farnesol at 16 μg/mL and EtBr at 1 μg/mL was 0.375, showing a good synergism; in contrast, the positive control EPIs all showed indifference, except for the combination of reserpine and EtBr, which showed a weak synergy (FICI = 0.5). These results indicate that farnesol is effective in blocking EtBr efflux like the reference EPIs used here. Farnesol not only increased the intrinsic susceptibility of *M. smegmatis* to EtBr but also showed exhibited a higher level of antimycobacterial activity and a better effect on the MIC of EtBr, compared with the reference EPIs. From the fluorometric method, farnesol significantly enhanced the accumulation of EtBr and the inhibition of efflux from cells preloaded with EtBr; this result suggested that farnesol acts as an EPI in mycobacterium. Noteworthily, in our study, farnesol also showed synergistic effect when combined with rifampicin in *M. smegmatis* mc^2^155, this furtherly confirmed that farnesol has efflux pump inhibition activity in mycobacterium.

Bacterial resistance to antibiotics typically involves drug inactivation or modification, target alteration, or a decrease in drug accumulation associated with a decrease in permeability and/or an increase in efflux [27]. It is now generally understood that EPs are becoming an important mechanism of resistance, both alone and in synergy with changes in the permeability of the outer membrane [28]. Several compounds have been reported to not only have EP inhibition ability but also affect membrane permeability, leading to disruption of cytoplasmic membranes. For example, chlorpromazine has also been reported to bind to membranes, increasing their permeability [8,29]; chlorpromazine may affect the integrity of the cell wall of a *M. smegmatis* strain expressing Tap (a protein from *Mycobacterium fortuitum*), allowing tetracycline to enter cells and to accumulate in large amounts and rendering Tap activity inefficient [30]. Another NorA EPI in *S. aureus*, totarol (a phenolic diterpene), may disrupt the membrane phospholipids of bacteria, leading to a loss of membrane integrity [31]. Brehm-Stecher *et al*. [13] indicated that the sesquiterpenoid compound farnesol can enhance permeability as a result of disruption of the cytoplasmic membrane [22]. Farnesol can disrupt the normal barrier function of the bacterial cell membrane, allowing the permeation into the cell of exogenous solutes such as EtBr and antibiotics [13]. The result of present study showed that farnesol has EP inhibition activity, including enhanced accumulation of EtBr and inhibition of efflux from cells preloaded with EtBr. Thus, similar to chlorpromazine and totarol, farnesol is an EPI, and it additionally affects membrane permeability through the disruption of cytoplasmic membranes.

In previous reports, farnesol has been shown to inhibit the proliferation of pancreatic cancer cells *in vitro* and *in vivo* [32], to affect the development of cariogenic biofilms by enhancing the cariostatic effectiveness of fluoride [33], to promote apoptosis and inhibit the growth of *Candida albicans* [34], and also to lower blood pressure in a hypertensive animal model [35]. Because farnesol and many other sesquiterpenoids are generally recognized as safe, these compounds may prove useful in combination with antimicrobials intended for use in foods or on food contact surfaces [13].

## Experimental

### Chemicals

Chlorpromazine, reserpine, verapamil, farnesol, rifampicin (Sigma-Aldrich), and carbonyl cyanide *m*-chlorophenylhydrazone (CCCP; Fluka) were dissolved in dimethyl sulfoxide (DMSO, Sigma-Aldrich, Steinheim, Germany). EtBr (Sigma-Aldrich) was dissolved in water. The components of the phosphate-buffered saline (PBS) were 0.01 M phosphate buffer, 0.0027 M potassium chloride and 0.137 M sodium chloride, pH 7.4. The chemical structure of farnesol is shown in Figure 1.

### Strain and culture conditions

*M. smegmatis* mc^2^155 ATCC 700084 (LGC Promochem, Teddington, Middlesex, UK) was used throughout the study. Bacterial cells were grown at 37 °C in Middlebrook 7H9 broth or Middlebrook 7H11 agar medium (Difco Laboratories, Detroit, MI), both supplemented with 10% oleic acid–albumin–dextrose complex (OADC). These strains were suspended in PBS to prepare a standard inoculum which was used to determine the minimum inhibitory concentrations (MICs). To ensure a invariable number of bacterial cells between experiments, the number of colony-forming units (cfu) in accord with aliquots of the inoculum was routinely calculated [9].

### Determination of antibacterial susceptibility

MICs of EtBr, chlorpromazine, reserpine, verapamil, CCCP, rifampicin and farnesol were determined by the broth microdilution method adapted from previous studies and in accordance with the NCCLS guidelines [9,36]. Briefly, *M. smegmatis* mc^2^155 was grown in 7H9/OADC-supplemented medium at 37 °C to an optical density at 600 nm (OD_600_) of 0.8. The bacterial cultures were diluted in PBS and the suspension turbidity was adjusted equivalent to a McFarland standard of 0.5. The adjusted bacterial suspension was diluted 100-fold to prepare the final inoculum used in the experiments, and 0.1 mL aliquots were added to each well of the 96-well plate. These wells already contained 0.1 mL of each agent at concentrations prepared from 2-fold serial dilutions in 7H9/OADC-supplemented medium. The plates were incubated at 37 °C after a period of time to measure the MIC results. Growth in the agent-free control-well was the evidence of the end of the test period. The MIC was defined as the lowest concentration of compound that inhibited visible growth.

### Checkerboard synergy assay

The activities of farnesol, in combination with EtBr, were evaluated in duplicate against *M. smegmatis* mc^2^155 by the checkerboard titration method in 96-well round-bottomed plates. Plates contained bacterial inocula (the same amount as was used in the above-mentioned drug susceptibility testing) and 2-fold serial dilutions of the compounds in total volumes of 200 mL of broth. The maximum and minimum concentrations of each diluted drug were at least a 4-fold increase of their MIC. Following seven days of incubation at 37 °C, the MICs of the drug combinations were read by visual inspection and the fractional inhibitory concentration index (FICI) for *M. smegmatis* mc^2^155 was calculated by the following equation [37]: FICI = FIC(A) + FIC(B) = C_A_^comb^/MIC_A_^alone^ + C_B_^comb^/MIC_B_^alone^, where MIC_A_^alone^ and MIC_B_^alone^ are the MIC values of drugs A and B when acting alone and C_A_^comb^ and C_B_^comb^ are concentrations of drugs A and B at the isoeffective combinations, respectively. The interpretation of the FICI was as follows: an FICI value of ≤ 0.5 represented synergy, an FICI value between 1 and 4 represented indifference, and an FICI value > 4 represented antagonism [38]. The same operation was conducted to evaluate the FICI between farnesol and rifampicin against *M. smegmatis* mc^2^155.

### Measurement of EtBr accumulation

This assay was performed according to modification of the previous methods by Rodrigues *et al*. [9] and Coldham *et al.* [19]. *M. smegmatis* mc^2^155 were grown in 7H9/OADC-supplemented medium (10 mL) at 37 °C to an OD_600_ of 0.8. The culture was centrifuged at 13,000 rpm for 3 min, the supernatant was removed, the pellet was washed once and then re-suspended in PBS. After adjusting the OD to 0.4, glucose and EtBr (to yield a final concentration of 0.4% and 1 μg/mL, respectively) were added to one set of microtubes containing 1.0 mL of bacterial suspension. Aliquots of 0.095 mL were distributed to replica sets of 0.2 mL microtubes and 0.005 mL EPIs were added. Replica tubes that without any EPI served as controls. The fluorescence was measured in real-time using a Victor3 1420 multilabel counter (Wallac Oy., Turku, Finland) equipped with a plate heater set at 25 °C. The fluorescence at excitation and emission wavelengths of 530 nm (bandwidth 5.0 nm) and 600 nm (bandwidth 10.0 nm), respectively, was measured at 3-min intervals for 45 min. Each experiment was repeated three times.

### Measurement of EtBr efflux

The effect of the agents on EtBr efflux activity was tested according to a modification of fluorescence techniques recently reported [9,19]. To ensure the maximum uptaking of *M. smegmatis* mc^2^155 cells with EtBr, conditions, as confirmed by cfu counting, were exhibited to lead to maximum accumulation of EtBr without causing any significant inhibition of growth. The assay was performed as follows: accumulation at 25 °C without glucose, the use of an EtBr concentration that caused a higher accumulation without compromising the cellular viability (4 μg/mL, corresponding to half the MIC) and the presence of verapamil at half its MIC (150 µg/mL, which was used to make sure that all the *M. smegmatis* mc^2^155 cells can load up maximum EtBr). The EtBr-loaded cells were centrifuged at 13,000 rpm for 3 min and re-suspended in PBS containing 0.4% glucose but no EtBr. After adjusting the OD_600_ to 0.4, aliquots of 0.095 mL were transferred to replicate 0.2-mL microtubes and 0.005-mL EPIs added. Replica tubes that without any EPI served as controls. EtBr efflux from the cells was monitored with a Victor3 1420 multilabel counter (Wallac Oy., Turku, Finland) equipped with a plate heater set at 25 °C. The fluorescence at excitation and emission wavelengths of 530 nm (bandwidth 5.0 nm) and 600 nm (bandwidth 10.0 nm), respectively, was measured at 3-min intervals for 45 min. Raw fluorescence values were analyzed using Excel (Microsoft) and subtraction of the appropriate control blanks. Each experiment was repeated three times.

## Conclusions

In summary, we show that farnesol not only increased the intrinsic susceptibility of *M. smegmatis* to EtBr but also presented relatively good antimycobacterial activity compared with the reference EPIs; farnesol possesses an EP inhibition ability that enhanced the accumulation of EtBr and the inhibition of efflux from cells preloaded with EtBr in *M. smegmatis* mc^2^155. In future studies, we plan to evaluate the underlying molecular mechanism of farnesol as an inhibitor for mycobacterial EPs.
